# Linguistic Features and Psychological States: The Case of Virginia Woolf

**DOI:** 10.3389/fpsyg.2022.823313

**Published:** 2022-03-16

**Authors:** Xiaowei Du

**Affiliations:** Department of Foreign Language, Huazhong University of Science and Technology, Wuhan, China

**Keywords:** psychological states, linguistic features, prediction, mental disorders, prevention

## Abstract

This study investigated the relation between psychological states and linguistic features with the case of Virginia Woolf. We analyzed the data from *The Diary of Virginia Woolf* and *Virginia Woolf: Biography* by automatic text analysis and statistical analysis, including stepwise multiple regression and Deep Learning algorithm. The results suggested that the significant linguistic features can jointly predict the psychological states of Virginia Woolf, including the emotional value of *anger*, the absolutist word “*everything*,” and the total of first-person plural pronouns. In addition, we found that the total use of first-person plural pronouns and the emotional value of *anger* were negatively related to mental health of Virginia Woolf. While the use of the absolutist word “*everything*” was positively related to mental health of Virginia Woolf. Meanwhile, we developed a model that can predict the psychological states of Virginia Woolf, with 86.9% accuracy. We discussed the findings and enumerated the limitations of this study at the end of the paper. The results not only complemented previous studies in the understanding of the relation between language and psychological health, but also facilitated timely identification, intervention, and prevention of mental disorders.

## Introduction

The relation between psychological states and linguistic features recently has attracted the interest of researchers. The motivation of such a line of research is that the psychological states of a person can be examined by his/her linguistic features (Nguyen et al., 2017) since the language of a person seems a reliable device to interpret his/her internal thoughts, emotions, and feelings (Herbert et al., 2019).

Some studies have explored the relation between psychological states and linguistic features. These studies are roughly categorized into two types. First, psychological states are assessed by the way people write about their experiences (Barnes et al., 2007; Tausczik and Pennebaker, 2010; Al-Mosaiwi and Johnstone, 2018; Kim et al., 2019). Second, psychological states are predicted by factors such as the diachronic changes of linguistic features in written texts (Rodrigues et al., 2016; Ziemer and Korkmaz, 2017; Eichstaedt et al., 2018; Boukil et al., 2019).

However, mixed findings were obtained regarding the relation between linguistic features and psychological states. First, psychological states are found developing closely related with different linguistic feature. For example, Barnes et al. (2007) found that the suicide texts reflect a linguistic trend in negative emotion words and death words. In contrast, Kim et al. (2019) showed a linguistic trend in modifier, numerals, first and second person pronouns, emotion words (positive, negative, sadness, and depression-related), and future tense verbs in the suicide texts. Second, the relation between linguistic features found in texts and psychological states are inconsistent. For example, Tausczik and Pennebaker (2010) found that people who consider suicide express more negative emotional words. Another example showed an opposite result that people who consider suicide express less negative emotional words (Barnes et al., 2007).

Therefore, the purpose of this study is to explore the relation between psychological states and linguistic features with the case of Virginia Woolf. The findings of this study may examine and complement previous studies and provide an approach to early identification, intervention, and prevention of mental disorders.

## Literature Review

In this section, we introduce linguistic features that have been used to recognize psychological states in previous studies and a computational method that has been used to analyze sentiments and emotions.

### Linguistic Features and Psychological States

Recent studies have shown that linguistic features such as personal pronouns, emotion words, absolutist words, color words, word count, and question marks may be used to identify psychological states.

The use of personal pronouns has served as a promising indicator of psychological states (Tausczik and Pennebaker, 2010). For example, Campbell and Pennebaker (2003) suggested that personal pronouns manifest psychological states from the perspective of a person’s social connection or social isolation. Specifically, an excessive use of first-person singular pronouns may be related to a high degree of self-involvement, while an increased use of the other pronouns may indicate improvement of social engagement (Cohn et al., 2004; Simmons et al., 2008). In addition, people use more first-person singular pronouns when in grief or depression or attempting suicide (Rude et al., 2004; Boals and Klein, 2005; Eichstaedt et al., 2018). Last, it is also found that fewer first-person singular pronouns may involve deceptive communications (Newman et al., 2003), while more first-person plural pronouns may indicate that he or she was in a happy marriage (Simmons et al., 2008).

Similarly, the use of emotional words is also correlated with psychological states (Barnes et al., 2007), though mixed findings were found regarding the relation between emotions and psychological states. The findings can be roughly categorized into three types. First, both positive and negative emotional words are related with mental health, and more negative emotional words and less positive emotional words reflect a less healthy mental state (Pennebaker et al., 2003; Kahn et al., 2007). Second, only negative emotional words are related with mental health, and more negative emotional words reflect a less healthy mental health (Kahn et al., 2007; Herbert et al., 2019). Third, more emotional expressions improve psychological states, such as positive emotional words, anger, or sadness (Rude et al., 2004; Graves et al., 2005; Barnes et al., 2007). It is worth noting that some studies stressed the impact of negative emotional expression on psychological states. Previous studies found that negative emotional words not only carry more information about mental health than positive emotional words (Garcia et al., 2012) but may also benefit psychological states (Lerner et al., 2003).

Absolutist words have also been employed to predict psychological states (Savekar et al., 2019) since absolutist words are believed to present an absolutist thinking (Al-Mosaiwi and Johnstone, 2018). Empirical studies have revealed that the absolutist thinking, a cognitive distortion related to extreme and rigid thoughts, may do harm to mental health (Savekar et al., 2019; Jones et al., 2020). More importantly, compared with negative emotional words, absolutist words may more accurately track the severity of the affective disorder (Al-Mosaiwi and Johnstone, 2018). To be specific, the language of suicidal ideation contains approximately 30% more absolutist words than that of anxiety and depression, and approximately 80% more than that of normal mental health (Al-Mosaiwi and Johnstone, 2018).

Other linguistic features such as color words, question marks, and word counts, may also be used to predict psychological states (Barnes et al., 2007; Tausczik and Pennebaker, 2010; Wadsworth et al., 2016). For example, Wadsworth et al. (2016) found that the use of *white* in Sylvia Plath’s poems increased significantly while the use of *gray*, *yellow*, *purple*, and *brown* decreased rapidly before Sylvia Plath committed suicide. Furthermore, Barnes et al. (2007) analyzed letters and diaries of suicidal youth and found that the use of question marks increased rapidly before their suicide. Last, some studies indicated that word counts may be related to psychological states since it reflects how engaged people are in the expression or how much information people produced (Alvarez-Conrad et al., 2001; Tausczik and Pennebaker, 2010; Wadsworth et al., 2016).

### Sentiment Analysis and Psychological States

Sentiment analysis has recently been used to study psychological states since it could take a step closer to understanding and anticipating an individual’s physiological states and needs (Al-Thubaity et al., 2018; Chatterjee et al., 2019; Moreno-Blanco et al., 2020). To be specific, sentiment analysis quantifies or extracts the polarity of sentiments, attitudes, emotions, and opinions of a given text (Cambria, 2016; Rendalkar and Chandankhede, 2018). More importantly, sentiment analysis broadly covers two dimension, namely, sentiment analysis in a narrow sense and emotion analysis (Lei and Liu, 2021). On the one hand, sentiment analysis in a narrow sense mainly identifies the sentiment polarities such as positive, negative, or neutral (D’Andrea et al., 2015; Lennox et al., 2020; Zucco et al., 2020). Some studies have applied sentiment analysis in a narrow sense to identify psychological states (Tausczik and Pennebaker, 2010). For example, Herbert et al. (2019) found that people express more negative emotions in their notes before committing suicide. Similarly, Eichstaedt et al. (2018) found that depressed people express more negative emotions, which contributed negatively to their psychological states. In addition, Rodrigues et al. (2016) detected positive or negative emotions of cancer patients by analyzing their texts in online communities to support their treatment.

On the other hand, emotion analysis focuses on recognizing a set of basic emotions, which may extend a more accurate and reliable detection of psychological states and feelings (Calabrese and Cannataro, 2016; Molina Beltrán et al., 2019). Previous studies have developed several versions of basic emotions. Specifically, Ekman (1993) proposed six universal emotions including anger, disgust, fear, sadness, joy, and surprise. Besides, Plutchik (1980) developed eight basic emotions such as anger, anticipation, disgust, fear, joy, sadness, surprise, and trust. Some studies have investigated the relation between basic emotions and psychological states (Desmet and Hoste, 2013; Ciullo et al., 2016). For instance, suicide individuals tend to express more emotional words such as anxiety, sadness, and depression in response to their feelings of anger or anxiety (Kim et al., 2019). Meanwhile, the use of joy or happiness words, revealing a sense of enjoyment, satisfaction, and pleasure, and these words are frequently used when an individual is in the situation of wellbeing, inner peace, love, safety, and contentment (Papapicco and Mininni, 2020). Additionally, the use of sadness words reflects the degree of social withdrawal or mood flattening, occurring with a higher frequency when an individual is most likely in grief, loss, frustration, depression, and suicide ideation (Barnes et al., 2007; Eichstaedt et al., 2018; Kim et al., 2019).

In fact, many studies have explored the relation between psychological states and linguistic features. However, a few limitations should be noted. First, most studies applied commercial tools such as Linguistic Inquiry and Word Count Program (LIWC) to analyze the psychological states of texts, which may limit the application of their findings in that such tools are not freely available. More importantly, these studies can only explore the linguistic features provided by LIWC, which may limit the comprehensiveness of the research. Second, few studies have examined the relation between psychological states and other linguistic features besides personal pronouns and positive and negative emotions (Wadsworth et al., 2016; Al-Mosaiwi and Johnstone, 2018). Third, previous studies yielded conflicting findings, such as different linguistic features that are related to psychological states (Eichstaedt et al., 2018; Kim et al., 2019), and a different relation between linguistic features and psychological states (Barnes et al., 2007; Tausczik and Pennebaker, 2010).

Therefore, this study aims to address the foregoing limitations and examine the relation between linguistic features and psychological states with the case of Virginia Woolf. More specifically, we use *Python* and *R* (version 3.6.0) to analyze the psychological states of texts, which can consider more linguistic features. In addition, this study examine linguistic features that are related to psychological states of Virginia Woolf. We also confirm the relation between linguistic features and the psychological states of Virginia Woolf. Finally, we aim to provide an equation to predict the psychological states of Virginia Woolf by linguistic features.

## Materials and Methods

In this section, we introduce the data, the methods for text analysis, and the statistical analysis in this study.

### Data

*The Diary of Virginia Woolf* (Bell, 1984) was used for text analysis in the present study for three reasons. First, Virginia Woolf was a British novelist and essayist regarded as one of the major modernist literary figures of the twentieth century (Boeira et al., 2016). Second, she had mental disorders and attempted suicide several times, and killed herself at the age of 59 (Androutsopoulou et al., 2019). Third, *The Diary of Virginia Woolf* (Bell, 1984) contains 1,577 diaries in total and covers 26 years, from 1915 until 4 days before death in 1941, except in 1916, Virginia Woolf didn’t write a diary due to a serious breakdown (Blodgett, 1989). It is a private text of self-presentation and provides more comprehensive and affluent information to reveal the relation between linguistic features and psychological states (Briggs, 2011; Androutsopoulou et al., 2019). We saved each diary piece as one text and obtained 1,577 texts in total.

*Virginia Woolf: A Biography* (Bell, 1972) was used in this study for three reasons. First, this book is more reliable than other biographies about Virginia as the writer is the nephew of Virginia Woolf. Second, the chronology record of this book is complete, from Virginia Woolf’s birth (1882) to her death (1912). More importantly, it is a chronology that records the stages of composition of Virginia Woolf’s books, her daily life, and especially her illness. We found the psychological states of Virginia Woolf are recorded including *normal*, *recovery*, *restlessness*, *anxiety*, *irritability*, *physical discomfort*, and *mental illness*. Thus, we extracted the information on psychological states of Virginia Woolf and the corresponding time for analysis.

### Data Search

In this study, we explored some linguistic features as shown in Table 1.

**TABLE 1:** The classification of variables.

The dependent variable	Psychological states	*1 normal, 2 recovery, 3 restlessness, anxiety*, *and irritability 4 physical discomfort, 5 mental illness*
The independent variables	Colors	*red, white, black, green, yellow, blue, purple, gray*
	Personal pronouns	*i, my, me, we, us, our, she, her, he, him, his, it, its, them, their, they, you, your, yours, i_my_me, we_our_us, you_your_yours, he_his_him, she_her, it_its, they_them_their*
	Absolutist word	*absolutely, all, always, entire, ever, every, everyone, everything, definitely, full, must, complete, completely, constant, constantly, whole never, nothing, totally, total absolutist words*
	Emotions	*anticipation, sadness, joy, trust, disgust, anger, surprise, fear*
	Question mark	
	Word counts	
	Sentiment value	

We calculated the sentiment and emotion values of each text with Jockers (2015) *syuzhet*, a lexicon-based sentiment analysis package in R (version 3.6.0). To help make the results more reliable, we first calculated the sentiment and emotion values at sentence levels and then computed the mean values of each text as the final value of the text. We chose the *Bing* lexicon (Liu, 2012) to calculate sentiment values and the *NRC* lexicon (Mohammad and Turney, 2013) to calculate the emotion values for the reason that the lexica contain comprehensive lists of sentiment and emotion words and have been widely used in sentiment and emotion research (Calabrese and Cannataro, 2016). With the *Bing* lexicon (Liu, 2012), each sentence was assigned a sentiment value, while each sentence was assigned emotion values from eight perspectives, i.e., anger, anticipation, disgust, fear, joy, sadness, surprise, and trust.

Besides the sentiment and emotion values, we also calculated other linguistic features of each text, such as the number of words, absolutist words, color words, personal pronouns, and question marks. First, following Al-Mosaiwi and Johnstone (2018), we counted the frequency of 19 absolutist words of a text. Second, we counted the frequency of all personal pronouns. It should be noted that we combined the frequency of subject personal pronoun, the object personal pronoun, and the adjective subject pronoun of each personal pronoun of a text (e.g., *i_my_me*) due to the very low occurrence of the pronouns. Third, following Wadsworth et al. (2016), we counted the frequency of each color word of a text, including *red*, *white*, *black*, *green*, *yellow*, *blue*, *purple*, and *gray*. The calculation of all the foregoing linguistic features was performed with a homemade Python script.

Last, due to different text lengths, we normalized the raw frequency of the linguistic features with the following formula.


$$\mathrm{Normalized}\,\mathrm{frequency}=\frac{\mathrm{Raw}\,\mathrm{frequency}}{\mathrm{Number}\,\mathrm{of}\,\mathrm{words}\,\mathrm{in}\,\mathrm{the}\,\mathrm{text}}\times 1,000$$

### Data Analysis

The stepwise multiple linear regression analysis was conducted to identify the relation between linguistic features and psychological state of Virginia Woolf, which was implemented with Venables and Ripley’s (2002) *MASS*, a powerful package for the statistical and graphical analysis in R (version 3.6.0). To be specific, the independent variables are the linguistics features of texts we extracted. Besides, the dependent variable is the psychological states of Virginia Woolf, which were expressed in numbers for identification and calculation (e.g., 1 represents normal). Our reasons for choosing the stepwise multiple linear regression are listed as follows. First, it identifies the independent variables that can serve as the influential predictors to identify the dependent variable, with the inclusion set at *P* < 0.05. More importantly, it also gains insight into the correlation between the influential predictors and the dependent variable. A positive value of regression coefficient presents a positive relation, and vice versa.

Then, we used the significant linguistic features to build a model for identifying psychological state of Virginia Woolf based on Deep Learning algorithms with the *RapidMiner Studio* (the educational version 9.7). We did so for the following reasons.

First, Deep Learning, as an advanced machine learning algorithm, involves in deciphering the hidden and more complex but meaningful phenomena within the data that the traditional statistical methods struggled to show (Wongso et al., 2017). In simple words, *Deep Learning* may build an automatic but more effective model for identifying psychological state of Virginia Woolf. Second, we can yield an objective evaluated criteria, namely, *accuracy*, with higher values for a better performance (Savoy, 2020).

In conclusion, we determined the significant linguistic features in identifying psychological state of Virginia Woolf, examined their relations, respectively, and built and evaluated the model for identifying the psychological state of Virginia Woolf.

## Results

The results show some interesting findings. First, we found the significant linguistic features that jointly predicted the psychological states of Virginia Woolf after examining a total of 65 linguistic features, respectively, in the stepwise multiple regression analysis. The significant linguistic features were the total usage of first-person plural pronouns, i.e., “*we_our_us*,” the emotional value of *anger*, and the absolutist word “*everything.*” The other linguistic features of this study had no significant effect on predicting the psychological states of Virginia Woolf (*p*-values were larger than 0.05).

Second, we found some correlations between the significant linguistic features and mental health of Virginia Woolf. Specifically, the total use of first-person plural pronouns (Coefficients = −0.007) and the use of emotional value of *anger* (Coefficients = −0.007) were negatively related to mental health of Virginia Woolf. In contrast, the use of the absolutist word “everything” (Coefficients = 0.074) was positively related to mental health of Virginia Woolf.

Last, we used the significant linguistic features to build a model for identifying psychological state of Virginia Woolf, which yielded 86.9% accuracy with Deep Learning algorithms.

## Discussion

Several points should be noted based on the results of the present study. First, the total use of first-person plural pronouns correlated negatively with the psychological states of Virginia Woolf. The reasons are listed as follows. First, the use of first-person plural pronouns serves as a mark of social connection, inclusiveness, and belongingness (Pennebaker et al., 2003). Second, fewer first-person plural pronouns serve as evidence of greater self-involvement and selfishness (Pennebaker et al., 2005). Thus, it was the degree of connection and belongingness reflected the psychological states of Virginia Woolf with the total use of first-person plural pronouns. Inconsistent with some previous research, the total use of first-person plural pronouns outperformed than other personal pronouns in predicting psychological states of Virginia Woolf. The result is most likely due to the property of diaries, Virginia Woolf always concerned and recorded her personal activities, thoughts, and feelings. Thus, the use of first-person singular pronouns occurred with a higher frequency but little variation; the use of second and third personal pronouns occurred with a lower frequency; only the use of first-person plural pronouns occurred with a higher frequency and variation. In addition, the total use of first-person plural pronouns highlighted the fluctuations in variation and ensured a significant statistical analysis.

Second, the use of anger words correlated negatively with the psychological states of Virginia Woolf. Many researchers have studied the relation between emotions and psychological states since emotions are one of the key aspects that characterize many mental health conditions (Pennebaker et al., 2003, 2005). However, mixed findings were obtained regarding the relation between emotions and psychological states. For example, Pennebaker et al. (2003) found a linear relation between positive emotion and mental health, and a curvilinear relation between negative emotion and mental health. Some studies found a negative relation between negative emotion and mental health (Kahn et al., 2007; Herbert et al., 2019). They stressed the negative emotions have a detrimental impact on psychological states. Thus, this study complements previous studies, which proved that the anger expression could predict and benefit the psychological states of Virginia Woolf. The reasons are listed as follows. First, anger is an interpersonal feeling, which typically emerges in a close relation (Fehr et al., 1999). Virginia Woolf was involved in a close connection with others and expressed anger, which is beneficial to her psychological health. In addition, angry expression triggers more optimism and shows a higher expectation for the future (Lerner et al., 2003). Last, anger expression helps people realize mistakes and promote problem-solving (Aarts et al., 2010).

Third, the absolutist word “everything” correlated positively with the prediction of the psychological states of Virginia Woolf. Consistent with previous research, absolutist thinking performs an unhealthy and inflexible way of thinking with more absolutist words (Savekar et al., 2019). As expected, the thought patterns of unhealthy psychological individuals are concerned with the feeling of absolutism. It might also signify that absolutist thinking harmed the psychological health of Virginia Woolf. The reasons are listed as follows. First, absolutist thinking disrupts emotion regulation, promotes emotional distress, and make people vulnerable to poor psychological and physical health (Al-Mosaiwi and Johnstone, 2018). Moreover, absolutist people are often perfectionists who see their values, goals, and outcomes as being right (Antoniou et al., 2017). They prefer anger, self-blame, and deprecatory thoughts (Savekar et al., 2019). It is of interest to note that the absolutist word “*everything*” outperformed than other absolutist words we extracted in predicting psychological states of Virginia Woolf. The possible reason is that the absolutist word “*everything*” represents an overly dichotomous thought. The dichotomous thought lacks tolerance and compromise, which may do harm to mental health (Jones et al., 2020). Moreover, “*everything*” served as an indefinite pronoun or premodifier in the diary of Virginia Woolf. Thus, the diachronic change of usage of “*everything*” was more significant than of other absolutist words.

Last, our study verifies the value of the proposed model for predicting the psychological states of Virginia Woolf. Consistent with previous studies, the results proved that a change of psychological states affects the words people used, and the words people used convey a great deal of information about their psychological health. More importantly, our study, similar to ample research, suggested that the model based on linguistic features for predicting psychological states may become increasingly feasible and more accurate. For example, Boukil et al. (2019) proposed an automatic system based on Deep learning algorithm to predict suicidal ideation through analyzing sentiment and feelings expressed on social media. Similarly, Nguyen et al. (2017) extract linguistic features and topics to discriminate depressed online communities from other groups based on machine learning algorithms. Particularly, it yielded 77.6% accuracy in the binary classification of depression vs. Bipolar by Lasso algorithms. Meanwhile, Eichstaedt et al. (2018) built a model with some linguistic predictors to identify depressed *Facebook* users, with a higher prediction accuracy (AUC = 0.72). In addition, de Ávila Berni et al. (2018) developed a model to identify texts proposed by suicidal individuals based on the Naïve-Bayes machine-learning algorithm. The model achieved a higher performance, with an accuracy of 80%. In all, we can develop our proposed model into a system or tool for assessing mental health, monitoring mental disorder, and preventing suicide.

## Conclusion

In this study, we explored the relation between linguistic features and the psychological states of Virginia Woolf, which generated three findings. First, the result confirmed the total use of first-person plural pronouns, the emotional value of *anger*, and the absolutist word “*everything*” can jointly predict the psychological states of Virginia Woolf. Second, we provided an effective model to predict psychological states of Virginia Woolf. We can further applied the model in various areas such as interventions, therapeutic protocols, and suicide preventions.

Two limitations of this study should be noted. First, we should consider more factors that affect psychological health to improve the explanatory power and usefulness of our equation, such as other linguistic features, social, environmental, economic, and political contexts. Second, we should examine whether our findings are generalizable in any way with more replicative studies on different people, language, and materials.

## Data Availability Statement

The original contributions presented in the study are included in the article/supplementary material, further inquiries can be directed to the corresponding author/s.

## Author Contributions

XD: investigation, formal analysis, methodology, validation, and writing–review and editing.

## Conflict of Interest

The author declares that the research was conducted in the absence of any commercial or financial relationships that could be construed as a potential conflict of interest.

## Publisher’s Note

All claims expressed in this article are solely those of the authors and do not necessarily represent those of their affiliated organizations, or those of the publisher, the editors and the reviewers. Any product that may be evaluated in this article, or claim that may be made by its manufacturer, is not guaranteed or endorsed by the publisher.
